# A Novel 3q29 Deletion in Association With Developmental Delay and Heart Malformation—Case Report With Literature Review

**DOI:** 10.3389/fped.2019.00270

**Published:** 2019-07-08

**Authors:** Adela Chirita Emandi, Andreea Iulia Dobrescu, Gabriela Doros, Capucine Hyon, Diana Miclea, Calin Popoiu, Maria Puiu, Smaranda Arghirescu

**Affiliations:** ^1^Discipline of Genetics, Victor Babeș University of Medicine and Pharmacy, Timișoara, Romania; ^2^“Louis Turcanu” Clinical Emergency Hospital for Children, Timișoara, Romania; ^3^IIIrd Pediatric Clinic, Pediatric Cardiology, Victor Babeș University of Medicine and Pharmacy, Timișoara, Romania; ^4^Département de Génétique Médicale, AP-HP, GHUEP, Hôpital Armand Trousseau, Paris, France; ^5^INSERM, UMRS 933, Hôpital Armand Trousseau, Paris, France; ^6^Sorbonne Universités, UPMC Univ Paris 06, Paris, France; ^7^Genetics Department Cluj-Napoca, Iuliu Hațieganu University of Medicine and Pharmacy, Cluj-Napoca, Romania; ^8^Discipline of Pediatric Surgery, Victor Babeș University of Medicine and Pharmacy, Timișoara, Romania; ^9^IIIrd Pediatric Clinic, Victor Babeș University of Medicine and Pharmacy, Timișoara, Romania

**Keywords:** 3q29, cytogenetics, intellectual disability, cardiac malformation, behavior

## Abstract

3q29 deletion syndrome is a rare disorder, causing a complex phenotype. Clinical features are variable and relatively non-specific. Our report aims to present an atypical, *de novo* deletion in chromosome band 3q29 in a preschool boy, first child of healthy non-consanguineous parents, presenting a particular phenotype (microcephaly, “full moon” face, flattened facial profile, large ears, auricular polyp, and dental dystrophies), motor and cognitive delay, characteristics of autism spectrum disorder and aggressive behavior. He also presented intrauterine growth restriction (birth weight 2,400 g) and a ventricular septal defect. SNP Array revealed a 962 kb copy number loss, on the chromosome 3q29 band (195519857–196482211), consistent with 3q29 microdeletion syndrome. FISH analysis using a RP11-252K11 probe confirmed the deletion in the proband, which was not present in the parents. Although the patient's deletion is relatively small, it partly overlaps the canonical 3q29 deletion (defined between *TFRC* and *DLG1* gene) and extends upstream, associating a different facial phenotype compared to the classic 3q29 deletion, nonetheless showing a similar psychiatric disorder. This deletion is different from the canonical region, as it does not include the *PAK2* and *DLG1* genes, considered as candidates for causing intellectual disability. Thus, narrowing the genotype-phenotype correlation for the 3q29 band, *FBX045* is suggested as a candidate gene for the neuropsychiatric phenotype.

## Established Facts and Novel Insights

### Established Facts

- 3q29 deletion syndrome is characterized by a variable clinical presentation, with mild to moderate intellectual disability, autism, gait ataxia, microcephaly, cleft lip and palate, chest wall deformity.
- Affected people also show psychiatric disturbances, including aggression, anxiety, hyperactivity, and bipolar disorder with psychosis.
- Cardiac malformations, were reported in 11/42 of people with 3q29 del.

### Novel Insights

- We report a *de novo* small deletion (<1Mb) on 3q29 in a patient with microcephaly, moon face, flat profile, global developmental delay, aggressive behavior, and ventricular septal defect.
- This cardiac malformation was reported previously in only two cases.
- This deletion is different from the canonical region, as it does not include the *PAK2* and *DLG1* genes, considered as candidates causing intellectual disability; thus, narrowing the genotype-phenotype correlation for the 3q29 band.
- *FBX045* is suggested as a candidate gene for the neuropsychiatric phenotype.

## Introduction

Rare diseases are complex pathologies that require a multidisciplinary team and a very rigorous clinical evaluation. Some have a distinct, nonetheless, non-specific phenotype that makes the clinical diagnosis difficult. 3q29 deletion syndrome is a rare condition, reported in 2001 (1) and characterized by a variable clinical presentation, with mild to moderate intellectual disability, autism, gait ataxia, a chest wall deformity and additional features, including microcephaly, cleft lip and palate, horseshoe kidney and hypospadias observed in very few patients. Congenital anomalies associated with this condition are cardio-vascular defects, gastrointestinal abnormalities, failure to thrive, and teeth abnormalities (1, 2).

The canonical deleted region (1.6 Mb) includes several genes (~22 genes) with important roles in brain and neurocognitive development (3). From those, *PAK2* and *DLG1* genes were considered as candidates for causing intellectual disability. The chromosome 3q29 band was identified as a risk factor for schizophrenia, autism, bipolar disorders (4), and other neuropsychiatric pathologies; however, the phenotype is not clearly defined (4–6).

3q29 deletion syndrome has an autosomal dominant transmission. The deletion is frequently associated with segregation of neuropsychiatric diseases in families (5, 6). The deletion presents with a variable phenotype; however, the neurocognitive abnormalities are presented in most patients (2).

We aim to present a case of atypical 3q29 deletion syndrome with a particular phenotype and intellectual disability in order to further refine the genotype phenotype relation.

## Clinical Presentation and Family History

The patient was born at 40 weeks of gestation, with a weight of 2,400 g, the first born from healthy non-consanguineous parents. The parents had normal intelligence and no reported behavioral issues. The patient's family history was unremarkable.

**Figure 1:**
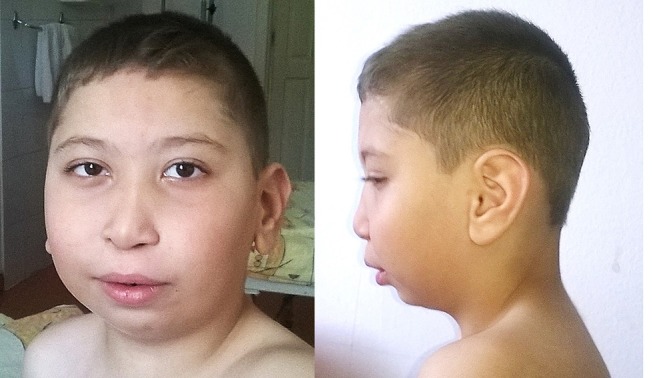
Patient face and profile at the age of 7 years.

At 7 years of age, the patient had a particular phenotype (Figure 1) with a “full moon” face, strabismus, antimongoloid slants, flattened facial profile, deviated septum, long philtrum, microretrognathia, dental dystrophies, high palate, large ears, auricular polyp, and a nasally voice. His body mass index was 19.3 kg/m^2^ (+2 standard deviations-SD) showing obesity. His height was 122.5 cm (+1 SD), within the normal range for boys at this age. At the age of 7 years he presented waddling gait and was not able to run. The clinical presentation of the patient in comparison to others reported in a 3q29 deletion registry (5), is presented in Table 1. The registry has the largest reported cohort in literature with 3q29 deletion.

**Table 1:** Clinical presentation of patient in comparison to phenotypic features, reported in a registry for 3q29 deletion (5) or by the literature review from Cox and Butler (6), arranged by decreasing frequency.

**Clinical feature**	**Previous reported % (no. with feature/total no. people)**	**Present case**
**NEUROPSICHIATRIC DEVELOPMENT (** **5** **)**		
Learning disability	98% (41/42)	+
Speech delay	59% (25/42)	+
Psychiatric disorder	28% (12/42)	+
Autism/autistic features	26% (11/42)	+
Anxiety disorder	19% (8/42)	
**CRANIO-FACIAL DYSMORPHISM (** **6** **)**		
High nasal bridge	72% (26/36)	–
Ocular abnormalities	58% (11/19)	+
Microcephaly	55% (18/55)	+
Short philtrum	51% (18/35)	–
Low-set, posteriorly rotated ears	43% (15/35)	–
Prominent or broad nasal tip/nose	40% (10/25)	–
Long narrow face	34% (12/35)	–
High-arched palate	33% (12/36)	+
Thin upper lip	30% (7/23)	–
Micrognathia	29% (7/24)	+
Abnormal brain MRI/CT	27% (3/11)	–
Brachycephaly	23% (6/26)	+
Frontal bossing	17% (4/23)	–
Facial asymmetry	17% (4/24)	+
Down-slanting palpebral fissures	17% (4/24)	–
Smooth philtrum	17% (4/23)	–
Cup-shaped ears	13% (3/24)	–
Large protruding ears	9% (2/23)	+
Broad nostrils	9% (2/23)	–
Long philtrum	9% (2/23)	+
Cleft lip/palate/submucous cleft	9% (3/32)	–
Up slanting palpebral fissures	8% (2/24)	+
**MUSCULOSKELETAL ABNORMALITIES (** **6** **)**		
Long/tapered fingers	37% (11/30)	–
Clinodactylous toes	32% (7/22)	–
Chest cavity deformity	29% (10/35)	+
Scoliosis	28% (5/18)	+
5th finger clinodactyly	19% (5/27)	–
Joint contractures	19% (4/21)	–
Abnormal palmar crease	17% (4/24)	–
Ligamentous laxity	11% (4/35)	–
Toe syndactyly	9% (2/23)	–
**GASTROINTESTINAL ABNORMALITIES (** **5** **)**		
Gastroesophageal reflux disease	43% (16/41)	–
Constipation	39% (9/41)	+
**GENITOURINARY DEFECTS (** **6** **)**		
Hypospadias	21% (3/14)	–
Horseshoe kidney	10% (2/21)	–
**OTHER FEATURES (** **5** **)**		
Abnormal teeth	66% (28/42)	+
Failure to thrive	39% (17/39)	+
Recurrent middle ear infections	32% (13/41)	–
Heart defects (PDA, ASD, and others)	26% (11/42)	–
**OTHER FEATURES (** **6** **)**		
Delayed walking	41% (14/34)	+
Low birth weight (<3rd percentile)	33% (10/30)	+
Ataxia gait/gait abnormality	38% (6/24)	+
Short stature	24% (7/29)	–
Abnormal skin pigmentation	14% (3/22)	–

*PDA, patent ductus arteriosum; ASD, atrial septal defect; No., number*.

The patient presented intellectual disability. At the age of 7 years, the patient's psychological development was comparable to that of a 2, 5 years old child. Additionally, he presented aggressive, sometimes uninhibited behavior and poor eye contact. Communication abilities included only hand gestures, and no words. He could interact with a mobile phone. The patient presented various anxieties (including a fear of dogs and stairs). He attended a normal kindergarten for 6 months, however, could not cope and thus, later attended services offered by a specialized center for intellectual disabilities.

His personal history showed delayed motor milestones. He started to walk around the age of 3 years and acquisitioned sphincter control after the age of 4 years. The history of illness included failure to thrive in the first 2 years, chronic constipation and repeated upper respiratory infections. He received treatment with Risperidone for his behavior, which showed a moderate response. Cardiologic evaluation at the age of 5 years, diagnosed the spontaneously closed ventricular septal defect.

The patient's imagistic brain evaluation, using MRI at 7 years of age was normal. Repeated EEG evaluations did not show significant changes.

## Methodology

### Clinical Evaluation

The patient was evaluated by clinical geneticists along with a multidisciplinary team. The mother completed a parent-report questionnaire (The Children Behavior Check List-CBCL) in order to evaluate her perception about the child's neuropsychiatric development.

The parents provided written informed consent to publish this case (including publication of images).

### Molecular Analyses

The genetic laboratory tests performed inlcuded: cytogenetic analysis (classic karyotype), SNP array using Infinium® OmniExpress-24 v1.2 Kit, ~710,000 Markers (scan performed using Iscan Illumina and GenomeStudio v2.0 software) and FISH analysis for patient and parents using the RP11-252K11 probe (localized to chr3:195907410-196071593 on build hg19, includes the *ZDHHC19, PCYT1A, SLC51A, TCTEX1D2*, and *TM4SF19* genes).

**Figure 2:**
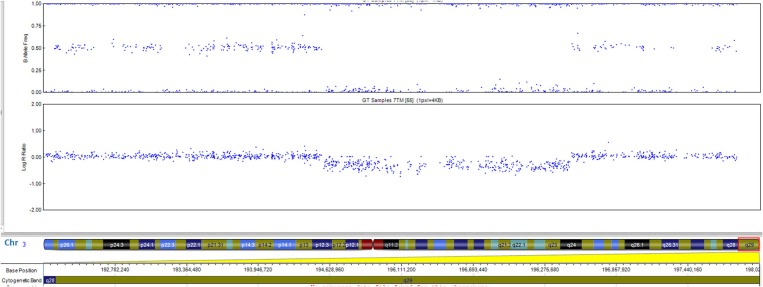
The 0.96-Mb deletion in the 3q29 band, arr[GRCh37] 3q29(195519857_196482211)x1 detected by whole genome SNP-array analysis and ideogram of chromosome 3. Morbid genes are depicted in red for the 3q29 band.

The patient has a normal male karyotype (46, XY). SNP array identified a 962 kb copy number loss of chromosome 3q29, between 195519857 and 196482211 (Figure 2). That region includes the following morbid genes *TFRC, PCYT1A, RNF168, TCTEX1D2*, and *CEP19*. Non-morbid genes included in the deleted region are: *TNK2, ZDHHC19, SLC51A, TM4SF19, UBXN7, RNF168, SMCO1, WDR53, FBXO45, PIGX*, and *NRROS*. The identified copy number variation was associated with 3q29 Microdeletion syndrome (OMIM 609425). No other significant genomic imbalances were detected. The 3q29 deletion was confirmed in the patient by FISH analysis using a RP11-252K11 probe. The parental genetic evaluation using the same FISH probe, was negative, thus, showing a *de novo* origin of the deletion. The FISH analysis in the parents cannot exclude small deletion/duplication/insertion in the targeting region. Gonadal mosaicism cannot be excluded and was considered in genetic counseling.

**Figure 3:**
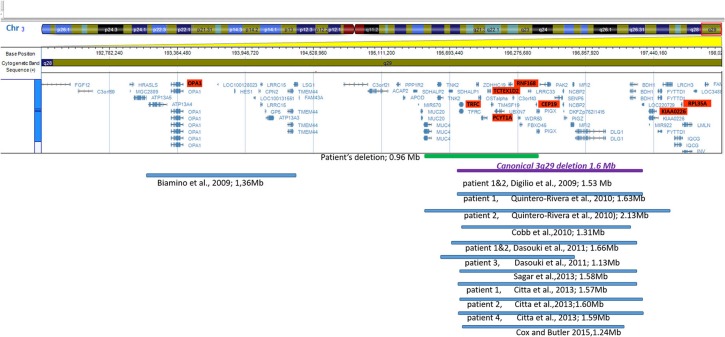
Ideogram of chromosome 3 with zoomed-in details of the 3q29 band shown with a schematic view of the genes involved in the deletion. Horizontal lines in blue show proportional representation of deletion size in our patient and previously reported deletions in other patients (in order of publication year). Deletion limits were converted to GRCh37/hg19 reference (also shown in Table 2). The ideogram included the deletions reported in the literature which were detected by array. Cases reported using the FISH method were not included.

A systematic literature search for similar reports was performed to have an overview of genotype and phenotype syndrome aspects. The findings are presented in Figure 3 (ideogram) and Table 2 (deleted limits).

**Table 2:** Deletion limits reported in literature using array platforms illustrated in Figure 3.

**References**	**Deletion limits** ***GRCh37/hg19***		
	**Start**	**End**	**Size Mb**
Patient 1 and 2 (7)	195,777,965	197,310,451	1.532
Patient 1 (2)	195,710,112	197,338,701	1.629
Patient 2 (2)	195,455,944	197,558,893	2.103
One patient (8)	195,771,743	197,085,623	1.314
Patient 1 & 2 (9)	195,689,972	197,358,134	1.668
Patient 3 (9)	195,601,025	196,732,851	1.132
One patient (10)	195,740,402	197,320,103	1.580
Patient 1 (11)	195,740,357	197,310,392	1.570
Patient 2 (11)	195,731,956	197,339,270	1.607
Patient 4 (11)	195,747,856	197,339,270	1.591
One patient (6)	195,788,299	197,033,296	1.245
One patient and 6 family members (12)	193,046,853	194,407,385	1.360
Our patient	195,519,857	196,482,211	0.962

### Discussion

Described initially as a 1.6 Mb deletion on THE long arm of chromosome 3 (1, 3), the 3q29 deletion syndrome was defined as a recurrent subtelomeric deletion (hg 19 coordinates 195,788,299–197,033,296) between *TFRC* and *DLG1* genes (6). Authors described families with 3q29 syndrome in several generations (11, 13). However, the present case is *de novo*, his parents do not present similar clinical or molecular abnormalities. The family history is not relevant for neuropsychiatric disorders.

The syndrome phenotype varies, from mild to severe, without very specific facial characteristics. Intellectual disability and psychiatric disorders are the most consistent findings (2). Glassford MR and collaborators have developed an online registry to summarize the phenotypic features of 3q29 deletion syndrome (5). To date, this is the largest cohort of patients with reported clinical data from 44 individuals. However, the data was self-reported/reported by family members and does not show deletion sizes to enable genotype-phenotype correlation. Authors highlighted the large clinical variability. Nonetheless, 64% of patients had a low birth weight and feeding problems, while ~30% of cases had neuropsychiatric disorders (anxiety, panic attacks, depression or bipolar disorder, and schizophrenia). The present case showed various anxieties already present at an early age. The patients in the registry also associated organ abnormalities. Cardiac defects were reported in 26% of cases, with the most frequently defect reported as ductus arteriosus and ventricular septal defect, a rare condition, reported only in two other cases (11, 14). The registry reported a high prevalence of gastrointestinal disorders (68%). In the present case constipation was one of the major issues for the family. The present case had rare clinical features (Table 1), reported in <20% of cases, such as large protruding ears (9%) and long philtrum (9%). Conversely, features frequently reported (in more than 40% of cases) were not identified, such as a high nasal bridge or long narrow face, however, these features could change with age.

The deleted region of the presented patient partly overlaps the canonical 3q29 deletion, associating a different facial phenotype compared to the classic 3q29 deletion, nonetheless showing a similar psychiatric disorder (Figure 1). *PAK2* and *DLG1* are autosomal homologs of the X-linked intellectual disability genes *PAK3* and *DLG3*. Haplosufficiency of *PAK3* or *DLG3* was previously associated with intellectual disability (14). Importantly, several transcription factors are mapped within this region, however their role in development is not completely understood. The patient reported by Krepischi-Santos et al. (15) had moderate intellectual disability and a particular phenotype caused by a small deletion of 1.0 Mb on the 3q29 chromosome, including gene *DIG1* (OMIM 601014) (5). Cobb's case, described in 2010 (8) was a 6 years old boy with a particular phenotype, autism spectrum disorders and ADHD but no intellectual disability, although the deleted region included morbid genes previously associated with intellectual disability (*DLG1* and *PAK2*) (8).

Out of the 11 non-morbid genes included in the deleted region of the patient, four (*TNK2, UBXN7, SLC51A, and FBX045)* have expressions in the brain. *TNK2* was reported (16), in a small family with autosomal recessive (AR), infantile onset epilepsy and intellectual disability. Functional studies were performed for the homozygous variant identified. Although our patient did not show seizures, this gene could potentially be considered relevant for the phenotype. The *SLC51A* gene was found to have a potential role of the organic solute transporter in brain dehydroepiandrosterone sulfate/pregnenolone sulfate homeostasis. This transporter was localized especially in steroidogenic cells of the cerebellum and hippocampus. The impact of transporters on neurosteroid homeostasis is poorly understood (17). The *UBXN7* gene was shown to be expressed in the brain, however its role is poorly understood at the moment (18). *FBX045* was suggested as a candidate gene for the neuropsychiatric phenotype by [Quintero-Rivera et al. (2)]. The *FBXO45* gene was also considered as a prominent candidate for mediating schizophrenia pathogenesis (19, 20), as the ubiquitin ligase F-box protein 45 is critical for synaptogenesis, neuronal migration, and synaptic transmission. While a genome-wide association study of coping behaviors suggests that FBXO45 is associated with emotional expression (21). Compared with other patient's deletion size and region involved in Figure 3, the closest is patient 3, described by [Dasouki et al. (9)], however patient phenotypic data was not available to the authors, except for the developmental delay and heart murmur, similar to our patient.

The function of the non-morbid *ZDHHC19, SMCO1, WDR53*, and *PIGX* genes is unclear or not relevant for the phenotype. Interestingly, the *NRROS, TM4SF19 (non-morbid), RNF168*, and *TFRC (morbid, AR)* genes were found to be related to the immune system, yet there were no apparent immune disfunctions in our patient or in the literature.

An Italian group (12) reported a 1.36 Mb deletion of the 3q arm, 193,046,853–194,407,385, outside of the known specific region for the classical 3q29 deletion syndrome, inherited from the mother and reported in several siblings, presenting with gastroesophageal reflux and milk protein intolerance as first clinical symptoms, later developing mild intellectual disability and endocrinological abnormalities. An overarching characteristic, described by Biamino was overweight, which was also observed in our patient. However, they do not share similar deleted regions. Bioamino et al. (12) suggested that the *HES1* gene could be causative for the obesity phenotype, while in our patient the *CEP19* gene (associated with Morbid obesity and spermatogenic failure-AR) in haploinsufficiency could play a potential role in our patient.

Aside from *CEP19*, other AR genes in the deleted region of the patient were: *TFRC*, associated with immunodeficiency 46 (OMIM 616740), *PCYT1A*, associated with Spondylometaphyseal dysplasia with cone-rod dystrophy (OMIM 608940), *TCTEX1D2*, associated with Short-rib thoracic dysplasia 17 with or without polydactyly (OMIM 617405), and *RNF168*, associated with RIDDLE syndrome (OMIM 611943). Considering the lack of association between patient phenotype and the symptoms of the associated pathology, these genes were not considered relevant for the phenotype.

The reported case provides additional clinical and molecular features that complete the phenotype and genotype of 3q29 Deletion syndrome.

A second hypothesis was considered for this particular case, however, the other CNVs observed in the SNP array analysis were benign. Also, the parental study showed *de novo* deletion 3q29. These arguments are supportive for the phenotype-genotype correlation. Nonetheless, the possibility of a second event, at the molecular level, cannot be excluded.

## Conclusion

We present the phenotype correlated with the smallest reported *de novo* chromosome 3q29 deletion (<1 Mb) in a patient with microcephaly, full moon face, flat profile, global developmental delay, aggressive behavior, and ventricular septal defect. This deletion is different from the canonical 3q29 deleted region, as it does not include the *PAK2* and *DLG1* genes, considered as candidates for causing intellectual disability, thus, narrowing the genotype-phenotype correlation for the region. *FBX045* is suggested as a candidate gene for the neuropsychiatric phenotype.

## Data Availability

This manuscript contains previously unpublished data. The name of the repository and accession number are not available.

## Ethics Statement

The parents provided the written informed consent to publish this case (including publication of images).

## Author Contributions

AC, AD, CP, MP, and SA contributed to patient clinical and genetic evaluation and the writing process. GD contributed to patient cardiological evaluation and the writing process. CH and DM contributed to patient genetic evaluation and the writing process.

### Conflict of Interest Statement

The authors declare that the research was conducted in the absence of any commercial or financial relationships that could be construed as a potential conflict of interest.
